# Supplementary material for: Comprehensive transcriptome analysis of peripheral blood unravels key lncRNAs implicated in ABPA and asthma
Source: PeerJ. 2021 Jun 24;9:e11453. doi: 10.7717/peerj.11453 (PMC8236232; doi:10.7717/peerj.11453)
Supplement: Supplemental Information 1 [file peerj-09-11453-s001.pdf]

Supplementary Information

Comprehensive transcriptome analysis of peripheral blood unravels key lncRNAs implicated in ABPA and asthma

Chen Huang[1†], Dongliang Leng[2†], Peiyan Zheng[3†], Min Deng[2], Lu Li[3], Ge Wu[3], Baoqing Sun[3*], Xiaohua Douglas Zhang[2*]

[1] Dr. Neher's Biophysics Laboratory for Innovative Drug Discovery, Macau University of Science and Technology, Macau, SAR, China

[2] CRDA, Faculty of Health Sciences, University of Macau, Taipa, Macau

[3] Department of Allergy and Clinical Immunology, State Key Laboratory of Respiratory Disease, National Clinical Research Center of Respiratory Disease, Guangzhou Institute of Respiratory Health, First Affiliated Hospital of Guangzhou Medical University, Guangzhou, Guangdong, China

[4] Stat Key laboratory of Quality Research in Chinese Medicine, Macau Institute For Applied Research in Medicine and Health, Macau University of Science and Technology, Macau, SAR, China

[†]These authors contributed equally to this work

*Correspondence: Xiaohua Douglas Zhang douglaszhang@um.edu.mo

Supplementary Methods

Transcriptome variations analysis

For SNPs and INDELs, Genome Analysis Toolkit (GATK) analysis pipeline was used. Briefly, the alignment outputs (bam files) were processed using Picard for marking duplicates and sorting alignment results. Then the indel realignment and base recalibration were performed for preparation of variant calling. Finally, SNPs and INDELs (vcf files) were detected and filtered. Variant annotation was conducted by SnpEff (4.3t) and annovar.

Then we used statistical hypotheses to investigate whether there were significant differences in the observed proportion of SNPs and INDELs among populations ABPA, asthma and healthy control. However, with the total samples is small, it seems not persuasive to find genetic differences based on the statistical hypotheses test. To settle this issue, we performed the statistical power analysis between different groups to filter the raw annotated variants. After deleting the variants with the power adequately less than 0.8, we conducted the hypotheses test on the remaining data. Concretely, we employed Fisher Exact probability test to compare the selected variation ratios between two different groups under the significant level $\alpha = 0.05$ since Fisher Exact probability test is reported to be appropriate for comparing proportions when sample size is small. P_{ABPA}, P_{asthma}, and P_{hc} were used to denote the proportions of variants

in populations ABPA, asthma and healthy control, respectively. To detect the difference between group ABPA and asthma, the corresponding hypotheses to test are as follows:

H_0: $P_{ABPA} = P_{asthma}$ $\quad versus \quad$ H_1: $P_{ABPA} \neq P_{asthma}$. If P-value $<$ 0.05, the null hypothesis H_0 is rejected, which means the proportions of variants are significantly different between group ABPA and asthma. Similarly, to investigate whether the proportions of variants are the same in populations ABPA and healthy control, asthma and healthy control, testing hypotheses were established accordingly.

For Simple sequence repeats (SSRs) detection, the Perl script of MISA–MicroSAtellite identification tool (http://pgrc.ipk-gatersleben.de/misa/) with default parameters was used, which could yield six distinct types of SSRs, including mono-nucleotide, di-nucleotide, tri-nucleotide, tetra-nucleotide, penta-nucleotide and hexa-nucleotide. We identified that each type of SSRs repeats 5~90 timesThen with fixed numbers of repeat, we exploited statistical hypotheses to investigate whether there were significant mean differences in the total numbers of SSRs with the statistical power equals to or granter than 0.8 among group ABPA, asthma and healthy control. For example, When the repeats = 5, we want to study the mean differences between the group ABPA and asthma, thus we have the following procedures to test the null hypothesis: H_0: $\mu_{ABPA} = \mu_{asthma}$ under significant level $\alpha = 0.05$,

- Use Shapiro-Wilk normality test to validate that the raw data from each of the two groups follows normal distribution;

- If the raw data follows normal distribution, use Welch t-test to obtain P-value (P_t), we can conclude that the mean values of group ABPA and asthma are significantly different when the $P_t < 0.05$;
- If the raw data is not normally distributed, use log-transformation to normalize the raw data. If normalized data follows the normal distribution, use Welch t-test to calculate P-value (P_t); If the normalized data is not normally distributed, it is recommended to use Wilcoxon rank test to obtain P-values (P_w) subsequently, then the mean values of group ABPA and asthma are considered significantly different if $P_w < 0.05$.
- Shapiro-Wilk normality test is not applicable when the raw data are identical in the testing groups. When this happens, use Wilcoxon rank test to acquire the determining P-value.

The whole statistic-test pipeline is visualized in Figure 3-A. Using the above statistical inference process, the testing hypotheses disclose whether the means are the same in populations ABPA vs health control, and populations asthma vs health control. Similarly, it is capable of doing similar analyses for each fixed-number SSR repeats.

Differential interaction network analysis

To perform differential interaction network analysis, an initial network was constructed for each group (ABPA, asthma and healthy controls). For each network, the correlation between

the differentially expressed transcripts and remaining transcripts (including mRNA-lncRNA, mRNA-mRNA and lncRNA-lncRNA) was assessed using Pearson Correlation Coefficient (PCC) algorithm based on the normalized expression profile via in-house R script. Then the difference of network between ABPA and health controls, as well as asthma and healthy controls were assessed by three criteria accordingly. This step generated three sub-networks for each group, including loss-of-function network ($|PCC_{healthy}| \geq 0.7$ & $P\text{-value}_{healthy} < 0.05$ & $|PCC_{disease}| \leq 0.30$), gain-of-function network ($|PCC_{healthy}| \leq 0.30$ & $|PCC_{disease}| \geq 0.80$ & $P\text{-value}_{disease} < 0.05$), and anti-function network ($|PCC_{healthy}| \geq 0.50$ & $|PCC_{disease}| \geq 0.50$ & $|PCC_{healthy} - PCC_{disease}| > 1$). Among these sub-networks, the interaction pairs which contained the transcripts significantly enriched in immune-related processes were retained. Subsequently, topological analysis was performed for each sub-network with cytoHubba V0.1 (36) plugin in Cytoscape V3.6.1 to detect key mRNAs and lncRNAs (hub nodes). 12 different algorithms of cytoHubba generated 12 hub node datasets. We selected the top 20 hub nodes from each algorithm after sorting by score. Finally, nodes with count frequencies higher than 2 in the 240-node set were regarded as highly confident hub nodes.

For the visualization of three immune-related sub-networks, more stringent filtering criteria were utilized as follows:1) loss-of- immune-related-function network in ABPA: $|PCC_{healthy}| \geq 0.95$ & $P\text{-value}_{healthy} < 0.05$ & $|PCC_{ABPA}| \leq 0.30$ & $|PCC_{healthy} - PCC_{ABPA}| > 1.00$; 2) gain-of-immune-related-function network in ABPA: $|PCC_{healthy}| \leq 0.30$ & $|PCC_{ABPA}| \geq 0.80$ & P-

$value_{ABPA} < 0.05$ & $|PCC_{healthy} - PCC_{ABPA}| > 1$; 3) anti- immune-related-function network in ABPA: $|PCC_{healthy}| \geq 0.70$ & $|PCC_{ABPA}| \geq 0.70$ & $|PCC_{healthy} - PCC_{ABPA}| > 1$; 4) loss-of- immune-related-function network in asthma: $|PCC_{healthy}| \geq 0.93$ & $P\text{-}value_{healthy} < 0.05$ & $|PCC_{asthma}| \leq 0.30$ & $|PCC_{healthy} - PCC_{asthma}| > 1.00$; 2) gain-of- immune-related-function network in asthma: $|PCC_{healthy}| \leq 0.30$ & $|PCC_{asthma}| \geq 0.90$ & $P\text{-}value_{asthma} < 0.05$ & $|PCC_{healthy} - PCC_{asthma}| > 1$; 3) anti- immune-related-function network in asthma: $|PCC_{healthy}| \geq 0.70$ & $|PCC_{ABPA}| \geq 0.70$ & $|PCC_{healthy} - PCC_{asthma}| > 1$.

For the integrated network of two diseases, we used permutation test to validate the significance of final hub nodes. Initially, we randomly perturbed all the interactions in the real network for 1000 times, which yielded 1000 pseudo networks. Likewise, we used 12 algorithms of cytoHubba to detect hub nodes and sort them from the top 20 hub nodes of each algorithm, the nodes with count frequencies higher than 2 in the 240-node set were used to compare to the real hub nodes.

Supplementary Figures and Tables

Figure S1. Basic statistics of variants annotated by annovar across 27 samples.

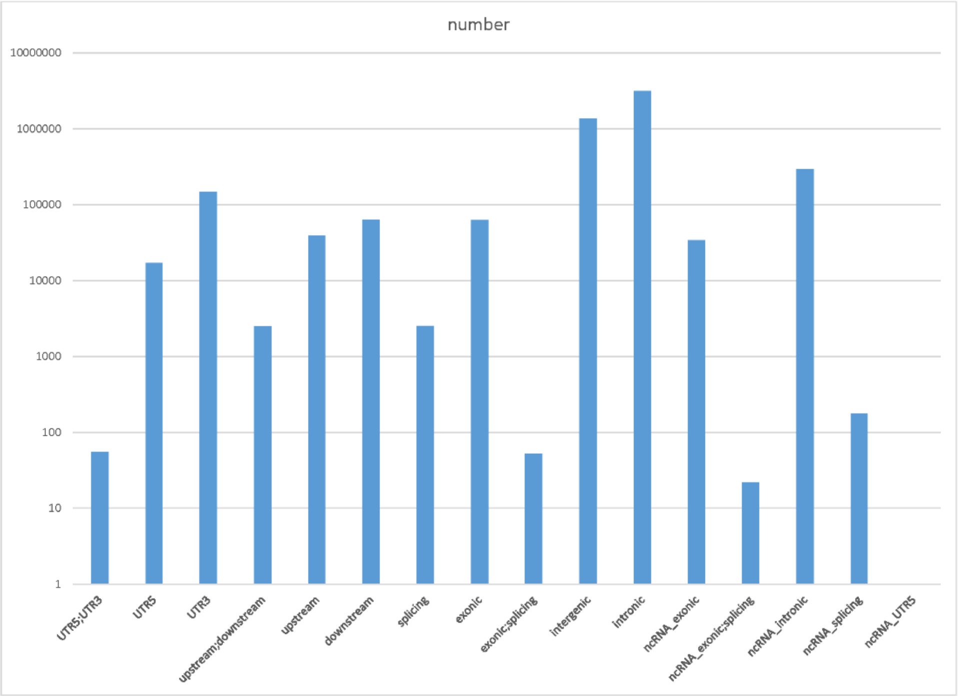

Figure S2. The mRNAs/lncRNAs architecture of the common immune-related functional network in ABPA and asthma. Three different dotted lines represents the common interaction pairs in three different types of networks between two diseases: loss-of-function network, gain-of-function network and anti-function network. All the interaction pairs were categorized into four immune-related groups according to their GO functional enrichment results.

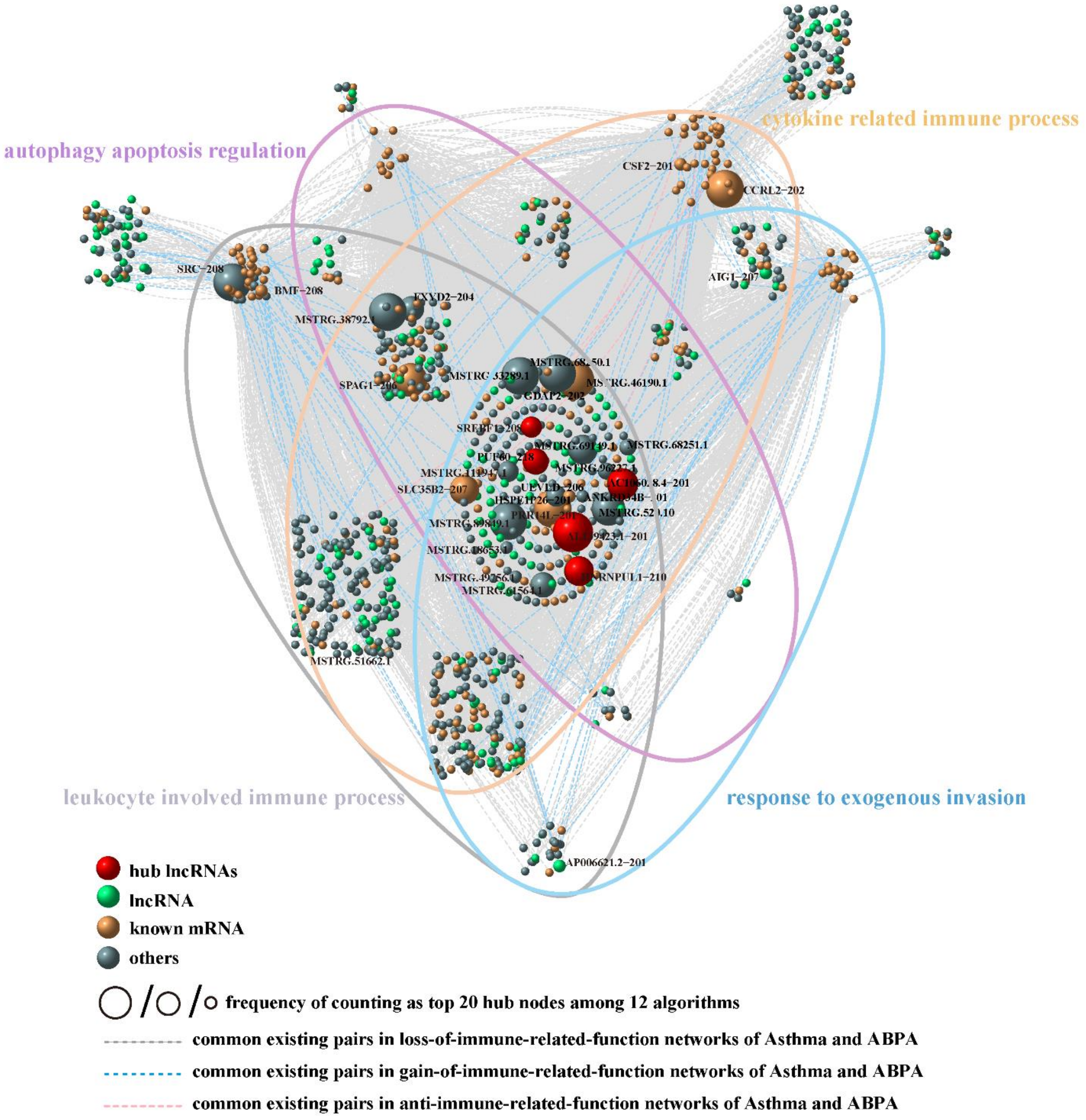

Table S1. Clinical staging of ABPA and asthma in the included patients.

**Group**	**Patient**	**Stage**
ABPA	AB_A1	Acute
	AB_A3	Acute
	AB_A4	Acute
	AB_A5	Exacerbation
	AB_A6	Acute
	AB_A7	Acute
	AB_B1	NA
	AB_B2	Response
	AB_B3	Acute
	AB_B4	NA
	AB_B5	Response
	AB_B6	acute
Asthma	AS_1	Exacerbation
	AS_2	Exacerbation
	AS_4	Exacerbation
	AS_5	Chronic
	AS_6	Exacerbation
	AS_7	Exacerbation
	AS_8	Chronic

ABPA staging: **Acute**, the patient is diagnosed as ABPA and features like elevated total as well as *Aspergillus*-specific IgE, radiological abnormalities, peripheral blood eosinophilia, and serum precipitins against *Aspergillus* are present; **Exacerbation**, any clinical and/or radiological worsening along with an increase in IgE level by >50% of baseline; **Response**, clinical as well as radiological improvement associated with at least 25% decline in serum IgE level at 8 weeks of therapy. Asthma staging: **Exacerbation**, progressive exacerbation of shortness of breath, coughing, wheezing, chest tightness, or a combination of the above symptoms, which can be quantified and monitored by lung function tests; **Chronic**, appearance of symptoms (wheezing, shortness of breath, chest tightness, coughing, etc.) at different frequencies and/or different levels every week. ABPA: Allergic bronchopulmonary aspergillosis.

Table S2. Detailed information of four GEO datasets for lncRNAs and mRNAs validation.

GEO dataset	Basic information (design, sequencing type, tissue)	Sample number	Validation Type	Validated RNA ID
GSE35571	asthmatics vs non-asthmatics, microarray, peripheral blood samples	131	mRNA	ENST00000327423
GSE473	asthmatics vs non-asthmatics, microarray, CD4+ cells from peripheral blood samples	175	mRNA	GDAP2-202
GSE31773	severe_asthmatic vs no_severe_asthmatic, microarray, CD4+ T cells from peripheral blood samples	40	lncRNA	ENST00000587128
GSE2125	Smokers vs nonsmokers vs asthmatics, microarray, alveolar macrophages	45	lncRNA	ENST00000595806

Table S3. Basic statistics of deep RNA sequencing data before and after processing.

Sample ID	group	Raw reads	Raw base	Q20/Q30 rate before filtration (%)	Clean reads	Clean base	Q20/Q30 rate after filtration (%)	Mapped reads	Mapped rate (%)
**AB_B1**	ABPA	140,639,534	21,095,930,100	99.75/95.02	136,247,784	19,750,718,680	100.00/97.94	91,626,316	84.22
**AB_B2**	ABPA	133,569,324	20,035,398,600	99.74/94.85	129,160,154	18,715,070,738	100.00/97.86	91,642,070	84.44
**AB_B3**	ABPA	128,610,498	19,291,574,700	99.53/92.76	121,402,388	17,242,172,432	100.00/97.93	91,268,320	77.30
**AB_B4**	ABPA	147,910,528	22,186,579,200	99.55/92.84	139,845,388	19,862,784,448	100.00/97.91	96,695,720	85.36
**AB_B5**	ABPA	142,796,748	21,419,512,200	99.57/93.51	136,401,698	19,163,991,174	100.00/97.47	118,973,560	87.32
**AB_B6**	ABPA	113,650,690	17,047,603,500	99.17/91.67	106,818,322	15,010,538,922	100.00/96.91	111,709,238	86.49

**AB_A1**	ABPA	110,664,956	16,599,743,400	99.28/92.04	104,892,772	14,816,854,370	100.00/97.15	83,357,458	68.66
**AB_A3**	ABPA	114,963,760	17,244,564,000	99.16/91.48	107,899,962	15,186,928,606	100.00/96.88	111,897,118	80.01
**AB_A4**	ABPA	116,145,080	17,421,762,000	99.35/91.29	108,797,132	15,233,514,448	100.00/96.73	110,207,150	80.80
**AB_A5**	ABPA	115,501,638	17,325,245,700	99.33/91.00	108,526,074	15,131,170,954	100.00/96.73	95,095,462	90.66
**AB_A6**	ABPA	126,609,044	18,991,356,600	99.25/91.26	118,072,948	16,277,108,454	100.00/97.09	88,640,084	82.15
**AB_A7**	ABPA	120,662,504	18,099,375,600	99.45/91.18	113,284,226	16,146,876,256	100.00/96.80	92,956,128	87.02
**As_1**	AS	157,397,512	23,609,626,800	99.72/94.70	151,907,042	21,927,907,770	100.00/97.81	96,572,818	84.03
**As_2**	AS	113,162,249	19,639,849,205	99.44/93.18	30,552,274	4,447,957,232	100.00/98.18	124,022,850	81.64
**As_4**	AS	123,229,582	18,484,437,300	99.29/90.89	114,920,908	16,094,794,128	100.00/96.56	26,269,506	85.98
**As_5**	AS	116,605,214	17,490,782,100	99.26/91.14	108,547,596	15,106,245,636	100.00/96.97	92,102,902	84.85
**As_6**	AS	131,709,112	19,756,366,800	99.28/91.41	123,041,210	17,133,966,112	100.00/97.06	103,081,148	83.78
**As_7**	AS	128,374,384	19,256,157,600	99.34/91.62	120,007,346	16,510,801,698	100.00/97.15	87,036,380	72.53
**As_8**	AS	139,095,408	20,864,311,200	99.51/91.84	131,143,780	18,768,426,602	100.00/97.03	115,931,322	88.40
**HC_1**	Healthy	151,397,014	22,709,552,100	99.77/95.26	146,931,478	21,349,804,756	100.00/98.02	131,185,634	89.28
**HC_2**	Healthy	144,001,122	21,600,168,300	99.75/94.59	139,158,410	20,110,822,056	100.00/98.01	119,763,746	86.06
**HC_4**	Healthy	164,485,936	24,672,890,400	99.64/92.81	154,144,788	22,045,298,224	100.00/97.93	136,237,618	88.38
**HC_19**	Healthy	139,564,308	20,934,646,200	99.18/91.60	131,105,060	18,417,212,460	100.00/96.87	113,465,560	86.55
**HC_22**	Healthy	137,440,108	20,616,016,200	99.12/91.30	128,643,068	18,096,858,012	100.00/96.80	108,655,078	84.46
**HC_23**	Healthy	101,018,208	15,152,731,200	99.23/91.55	93,494,474	13,204,896,592	100.00/96.86	71,753,898	76.75
**HC_25**	Healthy	116,540,484	17,481,072,600	99.40/92.30	110,611,886	15,471,599,552	100.00/97.31	48,355,508	43.72
**HC_28**	Healthy	134,612,054	20,191,808,100	99.43/92.92	128,187,652	18,228,363,894	100.00/97.33	109,757,760	85.62

Table S4. Basic statistics for all of variants occurred in three groups.

	Healthy	ABPA	AS
**SNP**	30,097,904	68,013,768	15,584,016
**INDEL**	3,685,704	8,093,796	1,916,432

Table S5. Basic statistics of variants occurred in genic region annotated by annovar.

Variant type	number
Synonymous SNV	26,911
nonsynonymous SNV	29,078
frameshift deletion	3,150
frameshift insertion	1,403
nonframeshift deletion	733
nonframeshift insertion	288
stopgain	727
stoploss	71
unknown	372

Table S6. Basic statistics for SSR occurred in ABPA, asthma patients and health controls.

SSR	Healthy	ABPA	AS
**mono-nucleotides repeats**			
**A/T**	1,059,279	1,729,494	749,768
**C/G**	9,476	14,557	6,381
**di-nucleotide repeats**			
**AC/GT**	114,831	191,895	82,069
**AG/CT**	45,352	76,238	30,609
**CG/CG**	1,345	2,185	977
**AT/AT**	37,774	66,099	28,202
**tri-nucleotide**			
**AGG/CCT**	6,177	9,970	4,523
**AGC/CTG**	5,617	8,641	4,204
**ACG/CGT**	116	192	93
**CCG/CGG**	8,360	12,345	5,884
**AAT/ATT**	18,578	30,059	12,371
**ACT/AGT**	604	1,016	440
**AAC/GTT**	20,089	32,701	13,866
**AAG/CTT**	4,250	7,144	2,906
**ATC/ATG**	3,670	6,030	2,656
**ACC/GGT**	2,638	4,610	1,869
tetra-nucleotide			
**AAAT/ATTT**	23,065	36,919	15,653
**AAAG/CTTT**	7,492	13,423	5,267
**AGAT/ATCT**	3,145	5,364	2,141
**AAGG/CCTT**	3,437	6,011	1,943
**AAAC/GTTT**	13,931	22,896	9,479
**others**	11,834	19,287	8,035
**penta-nucleotide**			
**AAAAC/GTTTT**	5,916	9,642	4,144
**AAAAT/ATTTT**	2,934	4,802	2,024
**AATGG/ATTCC**	1,938	3,818	967
**AAAAG/CTTTT**	1,340	2,288	916

**Others**	2,135	3,517	1,302
**hexa-nucleotide**			
**AAAAAT/ATTTTT**	116	203	88
**AGATAT/ATATCT**	110	171	78
**AAAAAC/GTTTTT**	530	885	381
**others**	969	1621	608

Table S7. Hub nodes of three sub-networks implicated in ABPA.

Transcript ID	Gene name	Type	Differential expression of regulation	frequency of counting as top 20 hub nodes among 12 algorithms	Type of sub-network
MSTRG.112085.1	MSTRG.112085.1	other	UP	9	anti
ENST00000462143	RGS3-210	mRNA	DOWN	9	anti
ENST00000309180	GPR171-201	mRNA	UP	8	anti
ENST00000233190	NDUFS1-201	mRNA	UP	8	anti
ENST00000455833	IFFO2-203	mRNA	DOWN	7	anti
ENST00000261250	C12orf4-201	mRNA	UP	7	anti
ENST00000256458	IRAK2-201	mRNA	DOWN	7	anti
MSTRG.31676.1	MSTRG.31676.1	other	UP	6	anti
ENST00000583313	RBM8A-204	mRNA	UP	6	anti
ENST00000562609[3]	GTF3C1-203	lncRNA	DOWN	6	anti
ENST00000518462[3]	REPIN1-216	other	DOWN	6	anti
ENST00000493352	MCM7-212	lncRNA	DOWN	6	anti
ENST00000399022	RPRD1A-202	mRNA	UP	6	anti
ENST00000380172	MTAP-201	mRNA	UP	6	anti
ENST00000373664	RBL1-202	mRNA	UP	6	anti
ENST00000286298	SLC26A2-201	mRNA	UP	6	anti
ENST00000378380	ANKEF1-201	mRNA	UP	5	anti
ENST00000267199	VPS33A-201	mRNA	UP	5	anti
MSTRG.91122.1[3]	MSTRG.91122.1	other	DOWN	4	anti
MSTRG.4929.1[+]	MSTRG.4929.1	other	UP	4	anti
MSTRG.18411.9[1]	MSTRG.18411.9	other	UP	4	anti
MSTRG.13021.1[+]	MSTRG.13021.1	other	UP	4	anti
ENST00000511180	NDUFA13-205	lncRNA	DOWN	4	anti
ENST00000496912[3]	TES-211	lncRNA	DOWN	4	anti
ENST00000487863[1]	ZNF808-206	mRNA	UP	4	anti
MSTRG.84188.1[1]	MSTRG.84188.1	other	UP	3	anti
MSTRG.66739.1	MSTRG.66739.1	other	DOWN	3	anti
MSTRG.6235.1	MSTRG.6235.1	other	UP	3	anti
MSTRG.58508.1	MSTRG.58508.1	other	DOWN	3	anti
MSTRG.22677.1	MSTRG.22677.1	lncRNA	UP	3	anti

MSTRG.20512.1	MSTRG.20512.1	other	DOWN	3	anti
MSTRG.110931.1[1]	MSTRG.110931.1	other	UP	3	anti
ENST00000637630	NRIP1-205	other	DOWN	3	anti
ENST00000574274	SLC43A2-207	other	DOWN	3	anti
ENST00000560141	TRIM69-207	other	UP	3	anti
ENST00000500949	OIP5-AS1-201	lncRNA	UP	3	anti
ENST00000473530	FCGR2C-204	other	UP	3	anti
ENST00000465812	IL1RN-207	other	DOWN	3	anti
ENST00000445132	CCR2-204	mRNA	UP	3	anti
ENST00000428670[+]	ATP2B1-204	mRNA	DOWN	3	anti
ENST00000381647[+]	C5orf51-201	mRNA	UP	3	anti
ENST00000374242	INIP-203	mRNA	UP	3	anti
ENST00000304625	RNASE2-201	mRNA	UP	3	anti
ENST00000248248	MON1B-201	mRNA	UP	3	anti
MSTRG.51584.8	MSTRG.51584.8	other	DOWN	8	lost
MSTRG.33289.1	MSTRG.33289.1	other	DOWN	8	lost
MSTRG.57029.1	MSTRG.57029.1	other	DOWN	7	lost
ENST00000525836	DSCAML1-202	mRNA	DOWN	7	lost
MSTRG.51682.11[3]	MSTRG.51682.11	other	DOWN	6	lost
ENST00000602230	BRD4-211	other	DOWN	6	lost
ENST00000327423	PRR14L-201	mRNA	UP	6	lost
MSTRG.96227.1	MSTRG.96227.1	other	DOWN	5	lost
ENST00000540010	TRAF1-202	mRNA	DOWN	5	lost
ENST00000465102	WIPI2-205	lncRNA	DOWN	5	lost
ENST00000369443[+]	GDAP2-202	mRNA	UP	5	lost
MSTRG.68251.1[3]	MSTRG.68251.1	other	DOWN	4	lost
ENST00000562599[3]	POLR2C-202	mRNA	DOWN	4	lost
ENST00000436788	MCM9-205	lncRNA	UP	4	lost
MSTRG.22673.1	MSTRG.22673.1	lncRNA	UP	8	gain
MSTRG.22670.12	MSTRG.22670.12	other	UP	8	gain
ENST00000360658	TLR9-201	mRNA	UP	8	gain
MSTRG.95648.1[1]	MSTRG.95648.1	other	UP	7	gain
MSTRG.20267.6	MSTRG.20267.6	other	UP	7	gain
MSTRG.20192.1	MSTRG.20192.1	other	UP	7	gain
ENST00000519975[1]	PCMTD1-204	lncRNA	UP	7	gain
ENST00000430881	TNFSF10-203	mRNA	UP	7	gain
MSTRG.90382.1[3]	MSTRG.90382.1	other	DOWN	6	gain
MSTRG.86322.1	MSTRG.86322.1	lncRNA	UP	6	gain

MSTRG.46039.1	MSTRG.46039.1	other	UP	6	gain
MSTRG.45137.1	MSTRG.45137.1	other	UP	6	gain
MSTRG.22670.8	MSTRG.22670.8	lncRNA	UP	6	gain
MSTRG.22674.1	MSTRG.22674.1	other	UP	5	gain
MSTRG.110932.1	MSTRG.110932.1	other	UP	5	gain
MSTRG.95646.1	MSTRG.95646.1	lncRNA	UP	4	gain
MSTRG.49797.1[2]	MSTRG.49797.1	other	UP	4	gain
MSTRG.37520.12	MSTRG.37520.12	other	UP	4	gain
MSTRG.22675.1	MSTRG.22675.1	other	UP	4	gain
MSTRG.109428.1	MSTRG.109428.1	other	DOWN	4	gain
MSTRG.52100.1	MSTRG.52100.1	other	UP	3	gain
MSTRG.28293.2[1]	MSTRG.28293.2	other	UP	3	gain
MSTRG.27653.1	MSTRG.27653.1	other	UP	3	gain
ENST00000428592	NEU4-210	mRNA	DOWN	3	gain

[+]: the transcript belongs to the common hub nodes of immune-related functional network in ABPA and AS.

[1]: the transcript exists in the cluster 3 of cluster analysis in Figure 5A.

[2]: the transcript exists in the cluster 3 of cluster analysis in Figure 5C.

[3]: the transcript exists in the cluster 3 of cluster analysis in Figure 5E.

Table S8. Hub nodes of three sub-networks implicated in Asthma.

Transcript ID	Gene name	Type	Differential expression of regulation	frequency of counting as top 20 hub nodes among 12 algorithms	Type of sub-network
ENST00000369763	LRIF1-201	mRNA	DOWN	11	anti
MSTRG.90604.7	MSTRG.90604.7	other	UP	9	anti
MSTRG.68296.7	MSTRG.68296.7	other	UP	8	anti
ENST00000347635	NUP50-201	mRNA	DOWN	8	anti
ENST00000562045	SYNGR3-202	mRNA	DOWN	6	anti
ENST00000560430	BMF-208	mRNA	DOWN	6	anti
ENST00000467084[3]	MRPS24-204	other	UP	5	anti
ENST00000263904	STAM2-201	mRNA	UP	5	anti
MSTRG.4929.1[+]	MSTRG.4929.1	other	UP	4	anti
MSTRG.25279.17	MSTRG.25279.17	other	DOWN	4	anti
MSTRG.13868.10	MSTRG.13868.10	other	UP	4	anti
MSTRG.11556.1	MSTRG.11556.1	other	UP	4	anti
MSTRG.113480.9	MSTRG.113480.9	other	UP	4	anti
ENST00000534295	SCUBE2-213	other	UP	4	anti
ENST00000513854[1]	HSD17B11-207	lncRNA	UP	4	anti
ENST00000475661	RN7SL105P-201	other	UP	4	anti
ENST00000344450[3]	DUSP22-201	mRNA	UP	4	Anti, lost
ENST00000341744	PMEPA1-202	mRNA	UP	4	anti
ENST00000341165	NBR1-201	mRNA	UP	4	anti
ENST00000300087	DCTN5-201	mRNA	UP	4	anti
ENST00000273398	ATP6V1A-201	mRNA	UP	4	anti
ENST00000270139	IFNAR1-201	mRNA	DOWN	4	anti
ENST00000164227[3]	BCL3-201	mRNA	UP	4	anti
MSTRG.60476.21	MSTRG.60476.21	other	DOWN	3	anti
MSTRG.58791.9[1]	MSTRG.58791.9	lncRNA	UP	3	anti
MSTRG.52480.5[3]	MSTRG.52480.5	other	UP	3	anti
MSTRG.19362.1	MSTRG.19362.1	other	DOWN	3	anti
MSTRG.13021.1[+]	MSTRG.13021.1	other	DOWN	3	anti
MSTRG.107593.1	MSTRG.107593.1	other	DOWN	3	anti
MSTRG.105806.1	MSTRG.105806.1	other	UP	3	anti
MSTRG.105004.1	MSTRG.105004.1	other	DOWN	3	anti
ENST00000598608	MED26-204	mRNA	DOWN	3	anti

ENST00000558841[3]	MAPK6-204	other	DOWN	3	anti
ENST00000473381	UBASH3A-204	mRNA	DOWN	3	anti
ENST00000466342[3]	SQSTM1-206	lncRNA	UP	3	anti
ENST00000405362	CACYBP-202	mRNA	UP	3	anti
ENST00000397128	PRKAA1-204	mRNA	DOWN	3	anti
ENST00000381647[+]	C5orf51-201	mRNA	UP	3	anti
ENST00000377294	ZKSCAN4-201	mRNA	UP	3	anti
ENST00000368130[1]	AIM2-202	mRNA	UP	3	anti
ENST00000358220[3]	WDR37-202	mRNA	DOWN	3	anti
ENST00000296435	CAMP-201	mRNA	DOWN	3	anti
ENST00000245817[3]	TNFSF9-201	mRNA	UP	3	anti
ENST00000218032	TLR8-201	mRNA	UP	3	anti
ENST00000559869	AC025580.2-201	lncRNA	DOWN	8	lost
ENST00000499292	AC104078.1-201	lncRNA	UP	8	lost
ENST00000399036	CCRL2-202	mRNA	UP	8	lost
ENST00000614509	AC106028.4-201	lncRNA	DOWN	7	lost
ENST00000507282	LPCAT1-204	lncRNA	DOWN	6	lost
ENST00000479735	ZEB2-221	other	UP	6	lost
ENST00000434686	GNB1-202	mRNA	DOWN	6	lost
MSTRG.7433.3	MSTRG.7433.3	other	UP	5	lost
ENST00000496420	PEX1-210	lncRNA	DOWN	5	lost
ENST00000262109	ERICH1-201	mRNA	DOWN	5	lost
ENST00000242208[1]	INHBA-201	mRNA	UP	5	lost
MSTRG.62218.1	MSTRG.62218.1	other	UP	4	lost
MSTRG.106338.1[3]	MSTRG.106338.1	other	UP	4	lost
ENST00000584377[3]	CSNK1D-222	lncRNA	UP	4	lost
ENST00000460254[3]	CERK-203	other	UP	4	lost
ENST00000369443[+]	GDAP2-202	mRNA	UP	4	lost
ENST00000263849	ZC2HC1A-201	mRNA	UP	4	lost
ENST00000252818	JUND-201	mRNA	DOWN	4	lost
MSTRG.53369.1	MSTRG.53369.1	other	UP	3	lost
MSTRG.520.10	MSTRG.520.10	other	DOWN	3	lost
ENST00000606802	AL139423.1-201	lncRNA	DOWN	3	lost
ENST00000564747	GTF3C1-205	lncRNA	UP	3	lost
ENST00000539605	ANKLE2-207	lncRNA	DOWN	3	lost
ENST00000497849	VIM-208	mRNA	DOWN	3	lost
ENST00000428670[+]	ATP2B1-204	mRNA	DOWN	3	lost
ENST00000265295[1]	SPDL1-201	mRNA	UP	3	lost

ENST00000258455	MRPS9-201	mRNA	UP	9	gain
MSTRG.108677.1[1]	MSTRG.108677.1	other	UP	8	gain
ENST00000379485	KBTBD6-201	mRNA	UP	8	gain
ENST00000373768	NDUFA8-201	mRNA	UP	8	gain
ENST00000369167	HIST2H2BF-201	mRNA	DOWN	8	gain
ENST00000397477	CPSF1P1-201	other	DOWN	7	gain
ENST00000374987	APEX2-201	mRNA	UP	7	gain
ENST00000314134	SLC35C1-201	mRNA	DOWN	7	gain
ENST00000272645	POLR2D-201	mRNA	UP	7	gain
ENST00000221132	TNFRSF10A-201	mRNA	UP	7	gain
MSTRG.95138.1	MSTRG.95138.1	other	DOWN	6	gain
MSTRG.5940.7	MSTRG.5940.7	other	DOWN	6	gain
ENST00000619039	PIP4K2B-204	mRNA	UP	6	gain
ENST00000375749	POFUT1-202	mRNA	UP	6	gain
ENST00000261772	AARS-201	mRNA	UP	6	gain
ENST00000370508	GOT1-201	mRNA	UP	5	gain
MSTRG.84882.11	MSTRG.84882.11	other	UP	4	gain
ENST00000378230	CEP104-202	mRNA	UP	4	gain
ENST00000216038	RTCB-201	mRNA	DOWN	4	gain
MSTRG.15670.1[1]	MSTRG.15670.1	other	UP	3	gain
ENST00000587128	AC006213.2-201	lncRNA	DOWN	3	gain
ENST00000557275	SYNE3-205	mRNA	UP	3	gain
ENST00000343629	TLDC1-201	mRNA	UP	3	gain

[+]: the transcript belongs to the common hub nodes of immune-related functional network in ABPA and AS.

[1]: the transcript exists in the cluster 3 of cluster analysis in Figure 5A.

[3]: the transcript exists in the cluster 3 of cluster analysis in Figure 5E.

Table S9. RNA-RNA interactions involved in hub nodes validated by GEO datasets.

Hub node	Target	PCC value in GEO dataset	GEO dataset
ENST00000595806	ENST00000323570	0.538	GSE2125
	ENST00000216484	0.689	GSE2125
ENST00000369443	ENST00000304222	0.666	GSE 473-GPL96
	ENST00000283147	0.559	GSE 473-GPL96
ENST00000327423	ENST00000426654	0.679	GSE 473-GPL96
	ENST00000261250	0.528	GSE 473-GPL96

Supplementary File 1: Fisher Exact probability test identified specific variants occurred in distinct groups.

Supplementary File 2: Two statistical methods identified significant differential enrichment of specific SSRs among those different samples.
